# Exploring Difference in Hand–Foot Coordination Ability Among Tennis Players of Different Sport Levels Based on the Correlation Between Lower-Limb Acceleration and Hand Grip Force

**DOI:** 10.3390/s25165152

**Published:** 2025-08-19

**Authors:** Yan Xiao, Jinghui Zhong, Yang Gao, Kebao Zhang

**Affiliations:** 1 Institute of Physical Education and Training, Capital University of Physical Education and Sports, Beijing 100191, China; ceciliaxiaoyan13@163.com; 2 School of Kinesiology and Health, Capital University of Physical Education and Sports, Beijing 100191, China; chungkingfai13@gmail.com; 3 School of Recreation and Community Sport, Capital University of Physical Education and Sports, Beijing 100191, China

**Keywords:** hand–foot coordination, accelerometry, grip force, tennis, performance analysis

## Abstract

Purpose: To quantify real-time hand–foot coupling in tennis and test whether the coupling pattern differs by playing standard. Methods: Fifteen nationally certified second-level male athletes and fifteen recreational beginners performed multi-directional swings, alternating forehand–backhand groundstrokes and serve-and-volley sequences while tri-axial ankle acceleration and racket-grip force were synchronously recorded in wearable inertial measurement units (IMUs). Grip metrics (mean force, peak force, force duration) and acceleration magnitudes were analysed with MANOVA and Hedges’ g effect sizes, followed by the Benjamini–Hochberg correction (α = 0.025). Results: Across tasks, athletes showed higher mean ankle acceleration (standardised mean difference, Hedges’ g) but 45% lower mean grip force (Hedges’ g = −1.28; both *p* < 0.01). The association between acceleration and grip metrics was moderate-to-strong and negative in athletes (r = −0.62 with mean grip force; r = −0.69 with force duration), whereas beginners exhibited moderate-to-strong positive correlations (r = 0.48–0.73). Conclusion: We quantified hand–foot coordination in tennis by synchronising tri-axial ankle acceleration with calibrated racket-grip force across three match-realistic tasks. Relative to beginners, athletes demonstrated an inverse coupling between ankle acceleration and grip-force metrics, whereas beginners showed a direct coupling, consistent with our purpose of quantifying coordination via synchronised wearable sensors.

## 1. Introduction

Effective stroke production in tennis depends on seamless energy transfer along the kinetic chain, linking the feet, trunk, and racket hand [1,2]. Empirical evidence shows that successful forehand, backhand, and serve actions require not only upper-limb coordination [3], but also precise phase locking between the trunk and arm [4], between the lower limb and trunk [5,6], and ultimately the lower and upper limbs [7], thereby forming an integrated whole-body system [8]. In racket sports, lower-limb impulses transmitted via ground reaction forces (GRF) initiate and modulate the kinetic chain that ultimately influences upper-limb and grip regulation [9,10].

Inter-limb coupling has been examined from multiple perspectives, defined as the task-dependent coordination in timing and magnitude between lower-limb drive and upper-limb/grip regulation within the stroke cycle [11]. Foot–ground studies indicate that stroke execution is lateralised: Sanjaya et al. reported side-specific plantar pressure patterns during dominant- versus non-dominant-side push-offs [12], while Lambrich & Muehlbauer demonstrated that grip technique systematically alters plantar pressure profiles during groundstrokes [13,14]. Upper-limb studies reveal complementary effects: Lock & Hsieh reported that a square stance elicited greater wrist-supination torque than an open stance (*p* = 0.032) [15], and Martin et al. indicated that fixing the back foot during the serve increased trunk and arm angular momentum [16]. Collectively, these findings confirm that lower-limb mechanics shape upper-limb output; nevertheless, most investigations have compared force-with-force variables and seldom quantify how rapid foot accelerations are coupled with dynamic grip-force modulation in real time.

In everyday coaching, emphasis is placed on rapid footwork and precise, phase-specific grip control [17]. Yet laboratory assessments typically approximate hand output using wrist-joint torque estimates derived from forearm kinematics, while overlooking direct measures of hand–muscle engagement. Grip behaviour in racket sports comprises two complementary modes: a power grip that generates impulse, driven primarily by forearm musculature with intrinsic-hand support, and a control grip that stabilises the racket during preparatory or quasi-static phases, governed chiefly by intrinsic-hand musculature [18,19]. We posit that performance advantages arise from an athlete’s capacity to alternate between these modes rhythmically and with accurate timing at the hand–wrist–forearm complex, thereby optimising energy transfer along the kinetic chain [20]. Because wrist torque alone cannot dissociate the contributions of power- and control-grip actions, there is a clear need for direct, high-resolution undergrip force sensing synchronised with lower-limb drive (e.g., ankle acceleration/GRF)—the central objective of the present study.

Hand–foot coordination can be conceptualised as the interaction between two distal elements of the kinetic chain: ankle acceleration (a proxy for lower-limb drive) and grip force (a proxy for upper-limb force application). Accordingly, we operationalise this construct using grip regulation indices—mean grip force and force duration—and tri-axial ankle acceleration magnitude as an index of lower-limb drive; these selections are supported by the racquet sport literature on flexible piezoresistive grip sensing [19,21,22]. A well-coordinated athlete is expected to adjust grip tension in concert with lower-limb drive and whole-body motion; conversely, mismatches between the two signals may indicate inefficient control. We therefore hypothesise that athletes will exhibit an inverse coupling—higher ankle acceleration co-occurring with lower and/or shorter grip-force deployment—whereas beginners will show weaker or direct (positive) coupling.

To test this hypothesis, we quantified real-time coupling between grip-force dynamics and ankle acceleration in two skill groups—15 nationally certified second-level athletes (hereafter, athletes) and 15 physical education majors (hereafter, beginners)—while they performed three match-realistic tasks: (i) multi-directional movement swings, (ii) alternating forehand–backhand strokes, and (iii) serve-and-volley sequences. The resulting metrics provide coaches with an objective indicator of hand–foot coupling and demonstrate the practical value of flexible piezoresistive sensors for on-court performance monitoring and broader sports technology applications.

## 2. Methods

### 2.1. Participants

Thirty right-handed male players comprised two groups: 15 nationally certified second-level athletes (n = 15; age = 18.0 ± 1.7 years; height = 1.771 ± 0.014 m; body mass = 67.3 ± 4.1 kg; match-play experience = 6.0 ± 1.7 years) and 15 beginners (physical education majors; age = 19.0 ± 1.1 years; height = 1.758 ± 0.031 m; body mass = 67.9 ± 2.4 kg; playing experience = 1.0 ± 1.1 years). All participants used a semi-western forehand grip and a two-handed backhand. Written informed consent was obtained for participation and for open-access publication of anonymised data and images. The study complied with the Declaration of Helsinki and was approved by the Ethics Committee of the North University of China (approval no. 202504014).

### 2.2. Apparatus and Experimental Procedure

Session overview: Each participant completed all procedures in a single session (60–75 min), with one participant on court at a time. Sessions were scheduled within consistent windows (09:00–12:00 or 14:00–17:00) to minimise diurnal variability. Task order was counterbalanced across participants. Rest intervals of ≥2 min were enforced between tasks, with ~30 s between trials; water was available ad libitum. Immediately after each task, participants reported Borg RPE to monitor exertion.

Warm-up and familiarisation (Step 1): Participants completed a standardised warm-up: 5–7 min light jogging and dynamic mobility (ankle circles, hip swings, walking lunges), followed by familiarisation with the instrumented racket (2 min dry swings plus 6–10 task-specific practice strokes per scenario). Familiarisation trials were excluded from analysis and served only to ensure comfort with the undergrip sensor.

**Figure 1:**
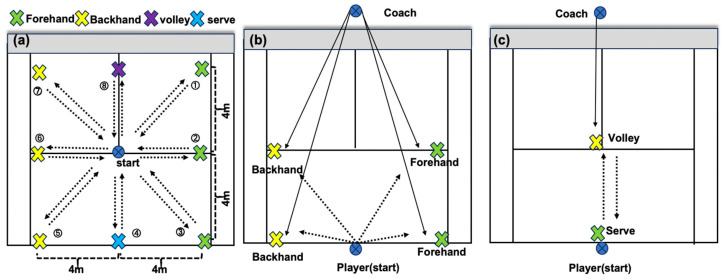
Testing process: (**a**) multi-directional movement swing; (**b**) alternating forehand–backhand; (**c**) serve–volley transitions.

Multi-directional swings (Step 2): Players moved to eight marked points clockwise and executed 16 full swings (8 paths × 2 cycles), returning to the start after each stroke (Figure 1a).

Alternating forehand–backhand (Step 3): A ball-feed sequence—baseline forehand, baseline backhand, mid-court forehand, mid-court backhand—was repeated until 16 valid strokes were obtained (Figure 1b).

Serve-and-volley (Step 4): From the right service court, players served and advanced to complete a forehand volley; four successful sequences were recorded (Figure 1c). Participants were instructed to perform each task at a self-selected maximal pace while preserving technique. Coaches fed balls only after athletes reset to the start mark to preserve rhythm. Verbal encouragement and ≥2 min rest separated tasks.

Pacing, fatigue, and order control: Participants were instructed to perform at a self-selected maximal pace while preserving technique. Coaches fed balls only after players reset to the start mark to maintain rhythm. Task order was counterbalanced, and rest periods (≥2 min between tasks; 30 s between trials) were enforced as above.

Feedback control: No augmented feedback (technical cues, knowledge of results, or real-time displays) was provided during data collection. Coaches standardised ball feeding and refrained from performance- or technique-related comments; only safety reminders and start/stop cues were permitted.

### 2.3. Outcome Acquisition Setup and Principle

**Figure 2:**
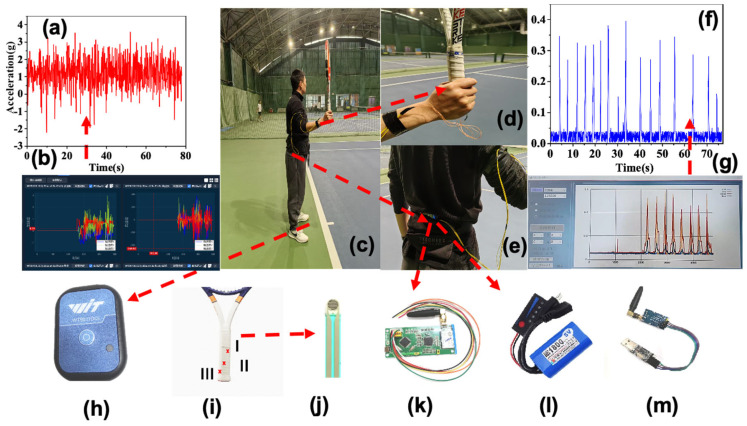
(**a**) Accelerometer (positioned at the participant’s ankle joint, covered by sports socks); (**b**) pressure sensor placement (located on the racket handle beneath the overgrip); (**c**) pressure sensor (data sensing and acquisition module); (**d**) pressure storage and transmission module; (**e**) power supply unit for the complete pressure sensor module; (**f**) computer-side receiver for pressure data; (**g**) schematic diagram of *Y*-axis acceleration; (**h**) JDU uppercomputer for acceleration data acquisition; (**i**) integrated device setup and participant wearing configuration; (**j**) participant demonstrating semi-western forehand grip; (**k**) wearing configuration of pressure sensor storage and transmission module and power supply; (**l**) schematic of participant’s hand average grip force variation; (**m**) JDU uppercomputer for complete pressure acquisition module.

Hardware overview: Grip force was measured with three MXene-based melamine sponge sensors (MMSS; NUC, Taiyuan, China) integrated beneath the overgrip on the racket handle (Figure 2b,c). A compact RF transmitter and battery powered the pads and streamed signals to a USB receiver attached to a laptop GUI that displayed the grip-current waveform in real time. Lower-limb drive was indexed using a tri-axial accelerometer (ActiGraph wGT3X-BT, Pensacola, FL, USA; 100 Hz) affixed at the ankle (Figure 2a).

Sensor placement and mounting: To accommodate habitual grip style, handle rotation between skills, and hand-size variability, the three undergrip pads were installed on three evenly spaced faces of the octagonal handle, beneath a fresh overgrip (Figure 2b,c). The accelerometer was positioned just superior and slightly anterior to the lateral malleolus of the dominant leg, with its long axis aligned to the tibial axis (arrows cranial). It was affixed with medical double-sided tape, secured by an elastic Velcro strap and overwrapped with athletic tape to prevent slippage. Placement marks and photographs ensured repeatability across tasks, and a brief tap test verified signal integrity.

Acquisition and synchronisation: Grip sensor output was streamed via RF to the laptop, while accelerometer data were transmitted via Bluetooth to the same host. Timestamps were aligned in the acquisition software, ensuring synchronous recording of grip current and ankle acceleration throughout each task.

Calibration and robustness: Grip current *I* (A) was mapped to force *F* (N) using a participant-specific linear bench calibration, *F = k · I + b*, with known loads. Linearity and precision were verified on the bench, and pre/post zero checks monitored drift. To mitigate handle rotation and contact-redistribution effects on the octagonal handle, instantaneous grip force was computed as the channel-wise maximum across the three calibrated pads, F(t) = max{F1(t),F2(t),F3(t)}. Prior racquet sport applications of flexible piezoresistive grip sensing support this approach [21,22].

Video assistance and event identification: A high-speed camera (i-Speed 5, iX Cameras, Rochford, UK; 250 frames·s^−1^) recorded trials to validate synchrony and to identify stroke events and phase timings (e.g., impact and preparatory phases).

Although both streams were time-stamped on the same host, they were ingested via separate pipelines (RF for grip, Bluetooth for accelerometry) rather than a single recording stack; thus, minimal residual temporal jitter cannot be entirely excluded. To mitigate this, we used window-based indices and validated key events with high-speed video.

### 2.4. Experimental Index

All outcome measures were computed for each participant across the three scenarios—multi-directional movement swings, alternating forehand–backhand strokes, and serve-and-volley sequences—with key parameters summarised in Table 1. Grip force was represented by the average grip force transmitted from three pressure sensors. Due to individual differences, such as different palm size and grip habits of the participants, the absolute values of the three grip-current values were selected to highlight the grip force of the athletes for analysis. Acceleration in a vertical direction was the *Y*-axis, parallel to the baseline of the tennis court was the *X*-axis, and parallel to the sideline of the tennis court was the *Z*-axis. The acceleration index tested in this study included gravitational acceleration. Because the acceleration due to gravity of the equipment (around 1 g) would produce some subtle changes in the X/Y/Z-axis as body posture changes, simply subtracting 1 g would not effectively filter out the influence of gravity on acceleration; the protocol maintained ecological validity while providing quantifiable metrics for coordination assessment. To avoid confusion with prior tasks, within-trial phases are denoted P1–P4: P1 Ready, P2 Approach (split-step/first move), P3 Stroke execution (plant-rotation-contact), and P4 Recovery (return to ready). Unless otherwise specified, indices labelled “Phases 2–4” in Table 1 were computed over the combined window from the first move through recovery (P2–P4).

### 2.5. Data Processing

Raw tri-axial acceleration (±16 g, 1000 Hz) and grip-force signals (±50 N, 1000 Hz) were synchronised via hardware trigger, resampled to 500 Hz, and low-pass filtered (4th-order Butterworth, 20 Hz). Trials with signal dropouts > 50 ms or motion artefacts > 3 SD from the participant’s mean were discarded (<3% of total). All variables were averaged across the three valid repetitions per task [19,21,22]. For each repetition we extracted (i) mean and peak ankle resultant acceleration, (ii) mean and peak grip force, (iii) grip-force duration (time above 5% of peak), and (iv) task-completion time. Hedges’ g was used for standardised effect sizes.

### 2.6. Statistical Analysis

Normality of model residuals and homogeneity of variance were examined using the Shapiro–Wilk and Levene tests, respectively. Box’s M was inspected for equality of covariance matrices. All tests were two-sided. A two-way repeated-measures MANOVA tested group (athletes vs. beginners; between-subjects) and task (multi-directional movement, alternating forehand–backhand, serve-and-volley; within-subjects) effects on the multivariate outcome vector (dependent variables as listed in Table 1). Pillai’s trace was reported for omnibus tests owing to its robustness to covariance inequality. For within-subject effects, Mauchly’s test assessed sphericity; when violated, Greenhouse–Geisser corrections were applied.

Significant multivariate effects were followed by Bonferroni-adjusted univariate ANOVAs (α = 0.05/2 = 0.025). Bootstrapped (5000 iterations) Pearson correlations quantified the coupling between ankle acceleration and grip metrics within each group; bias-corrected 95% confidence intervals (CI) were computed. Hedges’ g was labelled small (<0.5), medium (0.5–0.8), or large (>0.8). Using G*Power 3.1 with the current allocation (two-tailed tests), sensitivity analyses indicated that the study is adequately powered to detect effects of at least moderate magnitude, whereas small effects are unlikely to be detected with acceptable reliability. This framework is used to contextualise estimate precision throughout the Results. Analyses were performed in R 4.4.0 (packages: stats, car, effectsize, boot). Statistical significance was set at *p* < 0.05 unless otherwise specified.

### 2.7. Equipment Specifications

Chronograph (HS-80W, Casio, Tokyo, Japan); racket (Babolat Pure Drive, Lyon, France, 300 g); balls (Slazenger, Shirebrook, UK); MMSS grip sensors retained >95% sensitivity after 5000 load cycles (5.5 kPa) [23]; all hardware synchronised via a custom RF–Bluetooth hub.

## 3. Results

### 3.1. Multi-Direction Movement Swing

**Figure 3:**
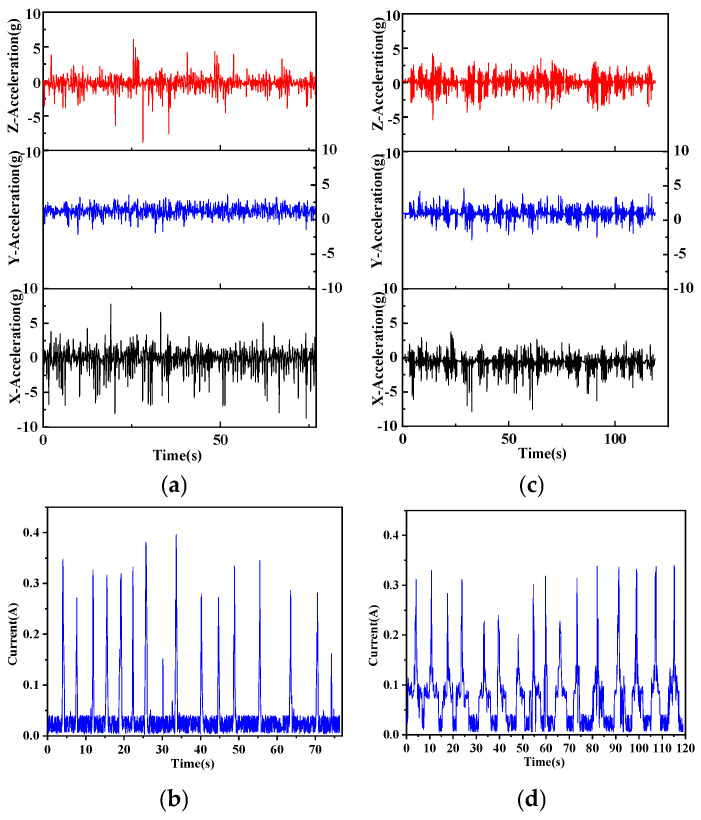
Grip force and lower-limb acceleration changes in multi-direction movement swing. Multi-directional movement swing—ankle acceleration and grip force. (**a**) *X*-axis ankle acceleration (m·s^−2^); (**b**) *Y*-axis ankle acceleration (m·s^−2^); (**c**) *Z*-axis ankle acceleration (m·s^−2^); (**d**) grip force (N).

As shown in Figure 3 and summarised in Table 2, athletes exhibited higher mean ankle acceleration than beginners along the *X*-axis (1.18 ± 0.17 g vs. 1.00 ± 0.15 g, *p* = 0.035) and *Y*-axis (1.27 ± 0.13 g vs. 1.11 ± 0.14 g, *p* = 0.047), with no difference along the *Z*-axis (0.79 ± 0.11 g vs. 0.73 ± 0.12 g, *p* = 0.189). Peak ankle acceleration was comparable along the *X*-axis (*p* = 0.213) but lower in athletes on the *Y*-axis (3.58 ± 0.55 g vs. 4.60 ± 0.67 g, *p* = 0.006) and higher in athletes on the *Z*-axis (8.86 ± 1.07 g vs. 5.41 ± 0.93 g, *p* = 0.004).

Within-group correlations indicated that, among athletes, mean ankle acceleration was moderately negative in association with mean grip force along the X-, Y-, and Z-axes (X/Y/Z: r = −0.56/−0.47/−0.50, *p* < 0.05 for all), and strongly negative in association with grip-force duration (r = −0.64 to −0.71, *p* < 0.01). Among beginners, the same variables showed moderate positive associations with mean grip force (r = 0.46/0.53/0.43, *p* < 0.05 for all) and strong positive associations with grip-force duration (r = 0.61 to 0.73, *p* < 0.01). Peak ankle acceleration was unrelated to any of the grip-force parameters in either group (*p* > 0.10).

### 3.2. Forehand-Backhand Alternating Stroking

**Figure 4:**
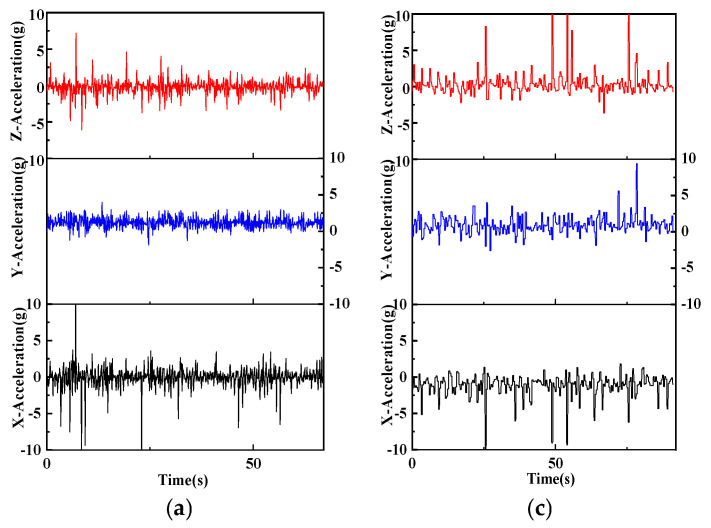

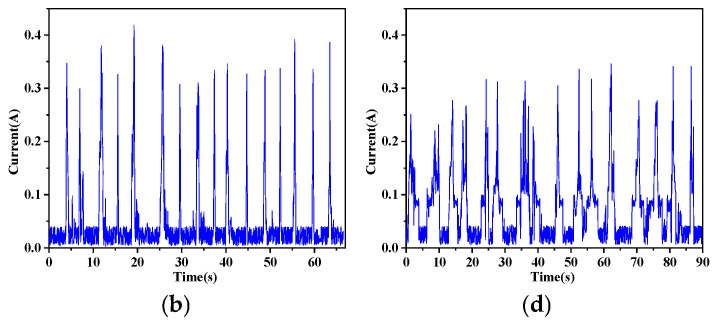
Variation in grip force and lower-limb acceleration in forehand–backhand alternating combination stroking situation. Alternating forehand–backhand—ankle acceleration and grip force. (**a**) *X*-axis ankle acceleration (m·s^−2^); (**b**) *Y*-axis ankle acceleration (m·s^−2^); (**c**) *Z*-axis ankle acceleration (m·s^−2^); (**d**) grip force (N).

As presented in Figure 4 and summarised in Table 3, mean ankle acceleration differed only along the *Z*-axis, where athletes recorded lower values than beginners (0.66 ± 0.09 g vs. 0.93 ± 0.12 g, *p* = 0.045); means along the X- and Y-axes were comparable (*p* = 0.621 and 0.120, respectively). For peak ankle acceleration, athletes showed higher peaks along the *X*-axis (12.35 ± 1.38 g vs. 10.47 ± 1.02 g, *p* = 0.037) and lower peaks along the Y- and Z-axes (3.95 ± 0.71 g vs. 9.38 ± 1.04 g, *p* = 0.001; 7.21 ± 0.88 g vs. 5.41 ± 0.93 g, *p* = 0.002, respectively).

Grip variables revealed no group difference in peak grip current (*p* = 0.735). However, athletes exhibited lower mean grip current (0.045 ± 0.007 A vs. 0.074 ± 0.009 A, *p* = 0.004) and shorter grip-force duration (0.62 ± 0.14 s vs. 1.65 ± 0.26 s, *p* < 0.001). Completion time averaged 67.1 ± 8.6 s for athletes and 91.2 ± 11.3 s for beginners (*p* < 0.001).

Within-group correlations indicated moderate negative associations between mean ankle acceleration and mean grip current in athletes (X/Y/Z: r = −0.41/−0.43/−0.57, *p* < 0.05 for all) and moderate positive associations in beginners (r = 0.48/0.49/0.51, *p* < 0.05 for all). Associations with grip-force duration were strong and negative for athletes (r = −0.74 to −0.66, *p* < 0.01) and strong and positive for beginners (r = 0.61 to 0.72, *p* < 0.01). Peak ankle acceleration was unrelated to any grip-force variable in either group (*p* > 0.10).

### 3.3. Serve–Volley Scenario

**Figure 5:**
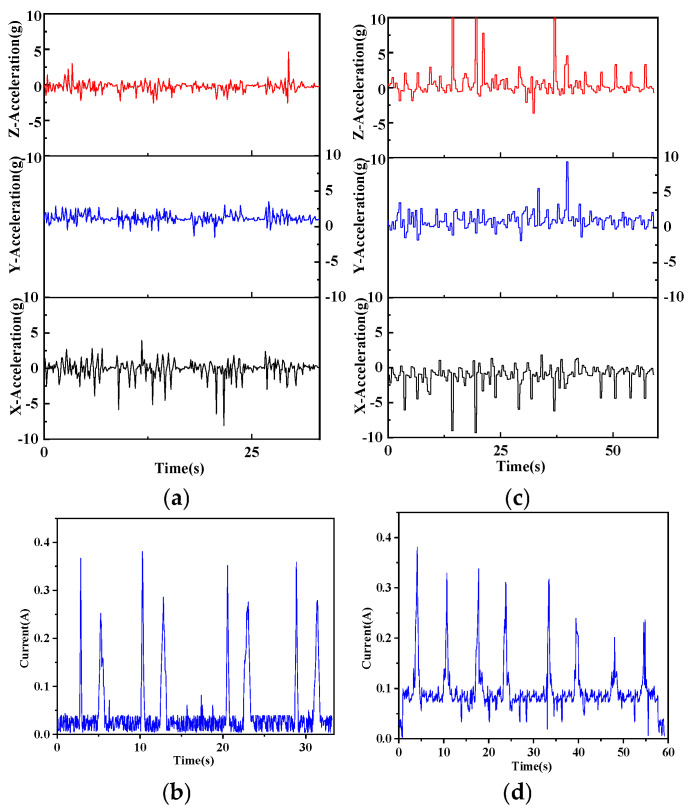
Variation in grip force and lower-limb acceleration in combination stroke situation of serve–volley. Serve-and-volley ankle acceleration and grip force. (**a**) *X*-axis ankle acceleration (m·s^−2^); (**b**) *Y*-axis ankle acceleration (m·s^−2^); (**c**) *Z*-axis ankle acceleration (m·s^−2^); (**d**) grip force (N).

As shown in Figure 5 and summarised in Table 4, group differences in mean ankle acceleration emerged only along the *Z*-axis, where athletes registered lower values than beginners (0.51 ± 0.10 g vs. 0.97 ± 0.11 g, *p* = 0.024). Mean X- and *Y*-axis accelerations did not differ (*p* = 0.253 and *p* = 0.960, respectively). Peak ankle acceleration diverged across all three axes: athletes showed lower peaks along the *X*-axis (8.14 ± 0.92 g vs. 9.40 ± 1.05 g, *p* = 0.039) and *Y*-axis (3.46 ± 0.70 g vs. 9.39 ± 0.98 g, *p* < 0.001) but higher peaks along the *Z*-axis (4.63 ± 0.83 g vs. 11.16 ± 1.12 g, *p* < 0.001).

Grip variables revealed no group difference in peak grip current (*p* = 0.915). However, athletes exhibited a lower mean grip current (0.043 ± 0.007 A vs. 0.094 ± 0.010 A, *p* = 0.002) and shorter grip-force duration (0.58 ± 0.14 s vs. 1.44 ± 0.23 s, *p* = 0.007). Completion time was shorter for athletes (33.2 ± 4.8 s) than for beginners (59.2 ± 7.5 s, *p* < 0.001).

Within-group correlations indicated moderate negative associations between mean ankle acceleration and mean grip current for athletes (X/Y/Z: r = −0.44/−0.47/−0.51, all *p* < 0.05) and moderate positive associations for beginners (r = 0.42/0.45/0.56, all *p* < 0.05). Corresponding relationships with grip-force duration were strong and negative in athletes (r = −0.70 to −0.63, *p* < 0.01) and strong and positive in beginners (r = 0.60 to 0.73, *p* < 0.01). Peak ankle acceleration was unrelated to any grip-force parameter in either group (*p* > 0.10).

## 4. Discussion

This study examined real-time hand–foot coordination in a match-realistic tennis setting by comparing athletes and beginners. The results revealed convergent differences: athletes demonstrated more economical lower-limb drive—expressed as a higher ankle acceleration magnitude together with lower and more precisely timed grip forces (shorter force duration)—consistent with the hypothesised inverse coupling pattern. Operationally, athletes achieved faster footwork while deploying only the necessary grip pressure, whereas beginners tended toward slower footwork and prolonged or excessive grip application. These findings refine performance evaluation by highlighting the link between lower-limb agility and upper-limb hand control, thereby offering coaches objective insight into how improving hand–foot coordination can enhance overall play. In addition, we leveraged a flexible piezoresistive undergrip pressure sensor to capture grip-force dynamics throughout the swing. This sensitive, lightweight device enabled time-resolved changes in grip pressure to be measured in real time, aligning with recent advances in wearable sensor technology for sports performance evaluation [21,22]. Grip-force metrics index the hand–racket contact load and its temporal deployment and are used here as measures of grip regulation rather than direct measures of intrinsic muscular force. By directly quantifying the ‘control-grip’ component, our results extend tennis biomechanics and support the concept that skilled movement involves a coordinated kinetic chain from the legs to the hand. The successful application of flexible pressure sensors in this context not only provides a novel perspective on performance assessment but also broadens the use of such wearable technology in sports science [21,22].

The experimental tasks were structured in ascending order of difficulty, beginning with multi-direction movement swings, followed by alternating forehand–backhand rallies, and culminating in a serve-and-volley sequence imposing the greatest coordinative demands. Throughout these tasks, athletes demonstrated more efficient lower-limb mechanics than beginners. Notably, accelerations along the lateral *X*-axis and the forward *Z*-axis reached higher amplitudes and occurred more frequently, whereas fluctuations along the vertical *Y*-axis remained minimal, as illustrated in Figure 3, Figure 4 and Figure 5. This combination allowed athletes to complete each drill in less time while selecting more economical movement trajectories. By contrast, beginners generated smaller and less regular acceleration bursts, leading to longer paths and slower execution. Accompanying this kinematic profile, athletes displayed a distinctive grip-force pattern: they maintained lower mean grip force and applied it within a shorter, more rhythmic time window, a trend again visible in Figure 3, Figure 4 and Figure 5. In other words, athletes maintained a steady, light grip during movement and timed force application precisely at ball impact, whereas beginners often applied greater grip force for longer than necessary. These observations align with prior research showing that lower-body action and stroke execution are closely coupled in tennis. For example, Lambrich & Muehlbauer [10] reported that elite players significantly shifted plantar pressure distribution when striving for higher shot speeds, underscoring the role of foot dynamics in stroke performance. Our findings also parallel recent sensor-based studies: Rigozzi et al. [24] found that recreational players exhibited substantially higher forearm muscle activity during the follow-through phase of the forehand than experienced players, suggesting a tendency to overgrip and sustain muscle tension longer into the stroke. Such tendencies are consistent with the inefficiencies observed in the beginner group’s hand–foot coordination. Consistent with this pattern, we found that overall ankle acceleration was moderately negative in association with grip force in athletes, whereas in beginners the association was moderately positive. This inverse relationship in athletes indicates the ability to relax the grip during intense footwork, thereby preventing unnecessary muscular tension, whereas the direct positive coupling in beginners suggests tightening of the grip as movement speed increases. These opposing correlation patterns underscore a key marker of advanced coordination: athletes can decouple and differentially regulate hand and foot actions when required, while beginners display a more undifferentiated coupling of effort across the limbs.

Several underlying mechanisms may explain why participants with higher skill levels demonstrated superior hand–foot coordination. First, from a neuromuscular control perspective, extensive training is thought to yield more stable and efficient neural control of movement. Athletes who have trained for years develop well-engrained motor programmes through repeated practice [25]. In this context, their central nervous systems have likely established a well-tuned feedforward command for tennis strokes, and the musculature and joints coordinate in a preplanned, reliable sequence. Proprioceptive feedback loops are also refined with long-term practice, helping to stabilise execution across repetitions. Second, in terms of sensorimotor feedback and inter-limb synchronisation, skilled coordination can be attributed to more precise internal timing and feedback-control mechanisms. In coordinated actions, the central nervous system uses feedback to align each limb’s motion with the intended rhythm, conceptually akin to each limb segment being governed by a local “position controller” that tracks a shared timing signal [26]. Thus, when the arms and legs must act in concert (e.g., propulsive push-off synchronised with the racket swing), an experienced player’s limbs remain phase-aligned to the common timing cue without requiring strong direct mechanical coupling [26]. By contrast, beginners have not yet established reliable sensorimotor coupling; their neural controllers for the hand and foot operate less in phase, impairing smooth coupling during complex movements. This discrepancy often manifests as off-timing—for example, grip force applied too early or too late relative to step timing.

Third, from a motor-coordination and kinetic-chain perspective, the differences can be framed in terms of how well each link in the movement chain is coordinated. Complex stroke execution depends on inter-segmental timing and scaling, with performance improving when segments act synergistically rather than in isolation [27]. According to the principle of motor abundance, the neuromotor system addresses the many degrees of freedom by organising joints and muscles into functional groupings or coordinative structures [25,26,27,28]. Athletes therefore appear to have developed an optimised coordinative structure for stroke production: neural circuits fire in a stable, accurate, and rhythmic sequence that activates the legs, trunk, arm, and hand. Practically, athletes can initiate a well-learned kinetic chain and execute it with minimal conscious intervention, resulting in consistently fluid movement. By contrast, beginners are likely still refining or differentiating their motor patterns. Their neural recruitment is not yet fixed into a stable order, yielding variability and inconsistency. This was evident as task complexity increased: beginners’ lower-limb acceleration patterns became more erratic and less goal-directed, and they often slowed or took extra steps to adjust. Simultaneously, their grip-force application extended over longer periods, with early onset (pre-impact) and prolonged post-impact maintenance, particularly during phases requiring direction changes or bursts of acceleration. In contrast, athletes maintained high-frequency, targeted leg drive and deployed grip force phasically at critical moments, relaxing rapidly between strokes. Such phasic, on–off use of grip force at the hand–wrist interface is likely a hallmark of an efficient kinetic chain in tennis. Our findings align with recent flexible sensor evidence: using a similar grip-pressure sensor, elite players exhibited shorter grip-force duration and greater grip-relaxation flexibility than intermediates, indicating that better performance correlates with the timely release of grip pressure [22]. This efficient hand usage, combined with the capacity to generate rapid lower-body movements, reflects a higher level of motor skill integration in advanced players. Notably, peak ankle acceleration and peak grip force were not temporally coincident and showed greater trial-to-trial variability than windowed summaries, leading to non-significant peak–peak correlations. By contrast, associations using mean grip force and force duration—and an exploratory time-aligned analysis (cross-correlation)—were qualitatively consistent with the group-distinct patterns (inverse in athletes, direct in beginners), without altering statistical interpretations.

It is important to situate hand–foot coordination as one facet of an athlete’s overall coordination capacity rather than an isolated two-limb phenomenon. Essentially, the harmonious interaction of feet and hands observed in tennis athletes is best understood as one manifestation of whole-body coordination ability [26,27,28,29]. In dynamic sports movements, the upper and lower body do not operate independently; for example, postural strategies involving the trunk and legs are intrinsically linked to arm actions [30]. Even routine activities rely on inter-limb coordination. Tasks involving both arms or multiple limbs—such as carrying objects, climbing stairs while using a handrail, or lifting heavy items—require the limbs to operate in concert, and any deficit in coordination can markedly reduce efficiency [30]. From a neurophysiological standpoint, coordinated limb movement is supported by communication between the left and right hemispheres. Because each side of the body is largely controlled by the contralateral hemisphere, effective coordination necessitates interhemispheric cross-talk via the corpus callosum and other pathways to produce unified movement patterns [31]. In tennis, superior hand–foot coordination therefore recruits the entire neuromuscular system: the legs, trunk, and arms must cooperate to generate and transfer force, and the brain must integrate signals from both hemispheres to synchronise grip action with footwork. Athletes appear to benefit from this integrative coordination—they coordinate step, torso rotation, and wrist action into a single fluid sequence. By contrast, beginners often show temporal disjunctions between segments, for instance initiating the swing before foot contact has fully stabilised or adopting excessive muscular stiffness that constrains arm mobility. Taken together, these points emphasise that improving hand–foot coordination is not merely a matter of training the hands and feet in isolation; it entails developing whole-body coordination, balance, and timing across segments.

Several limitations warrant acknowledgement. Scope: The study compared two skill tiers within tennis; cross-sport validation (e.g., squash, badminton) is needed to establish the generalisability of the coordination metrics. Instrumentation: The protocol focused on distal segments (ankle and hand). Future work should incorporate trunk sensing and full-body motion capture to position hand–foot coupling within the entire kinetic chain. Signal synchronisation relied on host-level time-stamping from separate RF/Bluetooth pipelines; therefore, small residual misalignment may persist despite video verification. Sample characteristics: The sample comprised male players only; sex-specific analyses may reveal additional coordination patterns. Although the sample size is typical for exploratory biomechanics, sensitivity analysis indicated adequate power for moderate effects but limited power for small effects. Non-significant results should therefore not be interpreted as evidence of no effect, and generalisation beyond the studied male cohort and two skill tiers should be performed with caution until larger, prospectively powered studies are conducted. Despite standardised pacing instructions, enforced rest intervals, and RPE monitoring, residual confounding by individual pacing strategy or latent fatigue cannot be completely excluded. Future work will include objective fatigue indices and preregistered, prospectively powered designs to better isolate technical proficiency from pacing-related influences.

In summary, our study demonstrates that hand–foot coordination distinguishes tennis skill levels and quantifies how this coordination is expressed. Athletes exhibited lower mean grip force, shorter force duration, and more precise temporal alignment between ankle acceleration and grip-force modulation, consistent with an efficient inverse-coupling strategy. By contrast, beginners lacked this pattern, showing slower footwork and less controlled grip use. A flexible piezoresistive undergrip sensor was instrumental in capturing these differences, offering an objective proxy for racket-hand control. Direct measurement of grip force adds a complementary dimension to evaluations of kinetic-chain efficiency that typically rely on joint moments and kinematics. The findings support the view that effective stroke production in tennis results from coordinated whole-body action. For coaches and practitioners, this work underscores the value of training programmes that integrate footwork drills with hand-control exercises to coordinate lower-body agility with upper-limb precision. Furthermore, embedding flexible sensor technology in on-court testing enables real-time monitoring of coordination metrics that were previously difficult to quantify. Overall, the study clarifies how elite-level coordination manifests between the hands and feet and opens avenues for improving training and advancing biomechanical assessment via wearable sensing.

## 5. Conclusions

This study quantified hand–foot coordination in tennis by synchronising tri-axial ankle acceleration with calibrated undergrip force across three match-realistic tasks. Relative to beginners, nationally certified second-level athletes (athletes) moved faster and more economically while applying lower and more precisely timed grip forces, resulting in an inverse association between ankle acceleration and grip-force metrics in athletes and a direct association in beginners. This contrast identifies a hallmark of advanced coordination—accelerating the feet while deliberately relaxing the hand—that avoids unnecessary tension and conserves effort during complex strokes. The derived wearable sensor metrics enable coaches to monitor whether grip pressure is released in step with explosive footwork and to support unobtrusive, on-court performance assessment, providing a practical, data-driven tool for training and biomechanical research.

## Figures and Tables

**Table 1:** Details and descriptions of selected experimental indices.

Index	Calculation Method	Meaning
Average lower-limb acceleration (m/s^−2^)	Average absolute acceleration at the ankle during Steps 2, 3, and 4 (X/Y/Z-axes), the sum of the acceleration in each direction, X/Y/Z, divided by the time.	The higher the number, the stronger the mobility.
Maximum lower-limb acceleration (m/s^−2^)	Maximum absolute acceleration at the ankle during Steps 2, 3 and 4 (X/Y/Z axes).	The higher the number, the stronger the mobility.
Average hand grip force (A)	The sum of average grip force in Steps 2, 3 and 4 divided by the completion time.	The lower the number, the more concentrated the grip power.
Maximum average hand grip force (A)	The maximum value of average grip force during the stroke in Steps 2, 3 and 4.	The higher the number, the stronger the grip.
Total grip force duration (s)	Total time when grip force occurs during the stroke in Steps 2, 3 and 4.	The lower the number, the more concentrated the grip force.
Completion time (s)	Completion time for Steps 2, 3 and 4 (s).	The lower the number, the higher the technical proficiency.

Note: A, ampere; s, second.

**Table 2:** Relation between grip force and lower-limb acceleration in multi-direction movement swing scenario (N = 30).

Index	Average Acceleration of Lower Limbs (m/s^−2^)			Maximum Acceleration of Lower Limbs (m/s^−2^)			Hand Grip Force (A)		Grip Force Duration Domain (s)	Completion Time (s)
	X	Y	Z	X	Y	Z	Average	Maximum	Average	
Beginners	1.00	1.11	0.73	7.93	4.60	5.41	0.071	0.388	1.28	119.2
Athletes	1.18	1.27	0.79	7.73	3.58	8.86	0.038	0.395	0.51	77.3
*p*	0.035	0.047	0.189	0.213	0.006	0.004	0.007	0.085	0.009	0.001
Significance	*	*	*	*	**	**	**	-	**	**

Note: A, ampere; s, second; *, significant difference (*p* < 0.05); **, very significant difference (*p* < 0.01); -, No significant.

**Table 3:** Relation between grip force and lower-limb acceleration in forehand-and-backhand alternating combination stroking situation.

Index	Average Acceleration of Lower Limbs (g)			Maximum Acceleration of Lower Limbs (g)			Hand Grip Force (A)		Grip Force Duration Domain (s)	Completion Time (s)
	X	Y	Z	X	Y	Z	Average Maximum Average	Average Maximum Average	Average Maximum Average	
Beginners	1.38	1.17	0.93	10.47	9.38	5.41	0.074	0.394	1.65	91.2
Athletes	1.46	1.20	0.66	12.35	3.95	7.21	0.045	0.407	0.62	67.1
*p*	0.621	1.211	0.045	0.037	0.001	0.002	0.004	0.735	0.000	0.000
Significance	-	-	*	*	**	**	**	-	**	**

Note: A, ampere; s, second; *, significant difference (*p* < 0.05); **, very significant difference (*p* < 0.01); -, No significant.

**Table 4:** Relation between grip force and lower-limb acceleration in combination stroke situation of serve–volley.

Index	Average Acceleration of Lower Limbs (g)			Maximum Acceleration of Lower Limbs (g)			Hand Grip-Current Value (A)		Grip Force Duration Domain (s)	Completion Time (s)
	X	Y	Z	X	Y	Z	Average	Maximum	Average	
Beginners	1.40	1.19	0.97	9.40	9.39	11.16	0.094	0.379	1.44	59.2
Athletes	1.64	1.19	0.51	8.14	3.46	4.63	0.043	0.382	0.58	33.2
*p*	0.253	1.059	0.024	0.039	0.000	0.000	0.002	0.915	0.007	0.000
Significance	*	-	*	*	**	**	**	-	**	**

Note: A, ampere; s, second; *, significant difference (*p* < 0.05); **, very significant difference (*p* < 0.01); -, No significant.

## Data Availability

All data comes from the References and is publicly available.
